# Removal of PET Microfibers from Simulated Wastewater Using Magnetic Nano-Ferric-Loaded Biochar: High Adsorption and Regeneration Performance

**DOI:** 10.3390/nano15120905

**Published:** 2025-06-11

**Authors:** Beisi Song, Nini Duan, Huaguo Xia, Yuan Li, Hongbin Xu, Ying Geng, Xin Wang

**Affiliations:** 1 School of Ecology and Environment, Zhengzhou University, Zhengzhou 450001, China; songbeisi@stu.zzu.edu.cn (B.S.); duannn@gs.zzu.edu.cn (N.D.); xhgzhizhu@stu.zzu.edu.cn (H.X.); xuhongbin_gy@zzu.edu.cn (H.X.); gengying@zzu.edu.cn (Y.G.); 2 Engineering Research Center for Water Emergency Response of Henan Province, Zhengzhou 450001, China; 3 Research Institute of Frontier Science, Southwest Jiaotong University, Chengdu 610031, China; xin.wang@swjtu.edu.cn

**Keywords:** magnetic biochar, microplastics, polyethylene terephthalate, adsorption, regeneration

## Abstract

Polyethylene terephthalate (PET) microfibers in effluent are difficult to remove using technology. In this study, a novel nano-sized iron-oxide-loaded biochar (FBC) with robust magnetic response characteristics was prepared by the impregnation–pyrolysis method and used for the removal of PET microfibers in simulated wastewater. The results showed that the removal efficiency of FBC on PET exceeded 91.69% over a wide pH range (4~9) and was barely affected by co-existing COD (15~500 mg/L) at an initial PET concentration of 1 g/L and FBC dosage of 3 g/L. The adsorption kinetics and isotherms indicated that the adsorption was more consistent with the pseudo-second-order kinetics (PSO) model and the Langmuir model, suggesting that the adsorption involved both physical and chemical actions. In addition, the maximum PET adsorption capacity expected by the Langmuir model reached 4500 mg/g, confirming the high adsorption performance of FBC. The characterization of FBC before and after adsorption indicated that PET was adsorbed mainly by the formation of Fe–O–PET bonds, π-π interactions, and hydrogen bonding. In addition, the FBC maintained a high PET removal efficiency of over 95.59% after four consecutive regeneration cycles. This study provides new insights into the efficient removal of fibrous microplastics from wastewater.

## 1. Introduction

Plastic pollution represents a pervasive environmental challenge on a global scale. According to Geyer et al. [1], approximately 6300 million metric tons (Mt) of plastic waste had been generated worldwide by 2015, of which only about 9% was recycled, 12% was incinerated, and the remaining 79% was either landfilled or discarded into the natural environment. Projections indicate that plastic waste deposits in landfill sites or natural environments may reach 12,000 Mt by 2050. Concurrently, annual plastic waste emissions are predicted to reach 53 Mt by 2030 under current disposal practices [2]. As a result of plastic pollution, substantial amounts of microplastics (MPs) are widely distributed across various environmental media including atmosphere, aquatic systems, and soils, even in living organisms, where they contribute not only to significant environmental pollution but also to adverse effects on the growth and health of flora and fauna [3]. The extensive distribution of MPs in natural environments has consequently posed a severe threat to the stability of ecosystems [3,4]. MPs in soil could alter its physicochemical properties, thereby influencing microbial activity and plant performance. Also, the ingestion of MPs and their derivatives by organisms in aquatic and soil environment could lead to toxicological effects such as growth inhibition, metabolic disorders, and even mortality [5]. What is worse, MPs have been detected in various human biological matrices, including brain and vascular tissues, infant placentas, and excreta, and MPs have been confirmed to exhibit toxic effects on the respiratory, digestive, and immune systems [6]. In addition, nano-sized MPs could cross the blood–brain barrier and accumulate in brain tissue, potentially impairing neurodevelopment and causing neurological damage [5,7]. Furthermore, MPs have been confirmed to interfere with gene expression, which may lead to an increased incidence of cancers associated with the lymphatic and hematopoietic systems. These latest findings underscore the significant threat posed by MPs to human health [7,8].

Wastewater treatment plants (WWTPs) receive MP pollutants through multiple methods, and their effluents have been identified as a major source of MPs in water environments [9,10,11]. Plastic debris generated from the fragmentation and degradation of plastic waste under natural processes such as photodegradation and weathering, along with synthetic fibers released from laundry wastewater, can enter urban sewer networks via domestic wastewater discharge and rainfall runoff. Although the majority of current urban wastewater treatment processes are capable of removing approximately 70–90% of MPs [10,11,12], a large amount of small-sized MPs (typically less than 0.5 mm in diameter) which escaped from treatment units, such as biological treatment and advanced oxidation units, could discharge into natural aquatic environments from the effluent of WWTPs [10,11]. It has been reported that the daily discharge of MPs in the effluent from WWTPs in China can reach 10^9^–10^10^ particles per liter [13]. Among them, polyester fibers, primarily polyethylene terephthalate (PET), constitute the predominant type of MPs found in influent and effluent, as well as sludge [14,15]. As a semi-crystalline thermoplastic polyester, PET exhibits high tensile strength, thermal stability, acid–alkali resistance, and pronounced electrostatic properties, rendering it highly resistant to biodegradation in the environment [16,17]. Consequently, PET is widely used in industries such as textiles. The microfibrous PET detected in treated effluents predominantly originates from processes such as laundry, carpet wear, and product packaging [18,19,20]. Due to its unique morphology, low density, recalcitrance, and high stability, microfibrous PET MPs are difficult to eliminate through conventional wastewater treatment technologies, such as the anaerobic–anoxic–oxic (A^2^O) process [12]. Although the concentration of PET microfibers in effluent was reported to be 83.16 ± 17.22 items·L^−1^, it may still have significant adverse impacts on receiving water bodies and aquatic organisms due to the large amount of discharged effluent [21].

Considering the emerging risk of MPs, the development of suitable technologies for the efficient removal of MPs from wastewater is essential for the mitigation of plastic contamination. Although water treatment technologies such as coagulation and sand filtration employed by WWTPs can remove MPs to some extent, these methods are associated with several issues, including limited removal efficiency, high costs, and the risk of secondary pollution [22,23]. For instance, membrane separation may lead to membrane fouling and has limited treatment capacity [24]. Coagulation processes may result in secondary contamination from coagulants and require proper disposal of MPs in the sludge [25,26]. Disposal costs associated with advanced oxidation processes are unacceptably high, and catalyst recovery and intermediate product toxicity are also issues to consider [27]. Moreover, the biodegradation of MPs such as PET is still at the laboratory research stage and has not yet been industrialized [28,29]. In comparison with other technologies, adsorption is more suitable for the mitigation of microplastic pollution in actual wastewater because it offers the advantages of low cost, operational simplicity, and minimal requirements for site conditions and equipment [26,30].

Agricultural waste-derived biochar is a promising adsorbent for MPs due to its large specific surface area, high durability, low cost, and easy availability [31]. However, conventional biochar suffers from limitations such as small particle size, low density, limited adsorption capacity, and difficulty in solid–liquid separation after adsorption [32,33]. Typically, separation of pollutant-adsorbed biochar requires filtration or centrifugation processes, during which the adsorbed pollutants may be desorbed and lead to secondary pollution [34,35]. These drawbacks compromise the economic and operational efficiency of using unmodified biochar for large-scale treatment of MP-contaminated wastewater. Recent studies have shown that biochar modified with transition metal ions, or their oxides, have a higher MPs removal capacity due to electrostatic interaction, chemical bonding, and surface complexation between modified biochar and MPs [35,36,37,38,39]. Furthermore, this kind of the modified biochar can be easily separated from wastewater and sludge by magnetic separation [33,35,40], which addresses the risks of MPs transfer and secondary contamination in sludge [41,42], thereby enabling the simultaneous removal of MPs from both sewage and sludge.

In this study, straw-derived biochar (BC) was used as the substrate, and a new-type magnetic nano-ferric-loaded biochar (FBC) composite adsorbent with high MPs removal efficiency and magnetic separation capability was synthesized through an impregnation–pyrolysis method. The adsorption performance and mechanisms of FBC for PET microfibers in wastewater were investigated, and the regeneration performance of FBC was also estimated. The results of this study provide a promising approach for the low-cost and efficient removal of PET microfibers from effluent.

## 2. Methods and Materials

### 2.1. Chemicals and Reagents

The biochar (BC) used in this study was derived from straw, with a bulk density of 400–450 g/L, a specific surface area of 1000–1500 m^2^/g, an ash content ranging from 5% to 10%, and a carbon content exceeding 95%. The PET microfibers used have a diameter of approximately 15 μm and a length of 38 mm. The main reagents used in the experiments and research processes, such as FeCl_3_·6H_2_O, FeCl_2_∙4H_2_O, NaOH, HCl, C_2_H_5_OH, and C_6_H_12_O_6_, are all of analytical reagent grade. The pH of the experimental solutions was adjusted by using HCl and NaOH.

### 2.2. Preparation of FBC

A mass of 4 g of FeCl_3_∙6H_2_O and 5.4 g of FeCl_2_∙4H_2_O were accurately weighed and dissolved in 100 mL of deionized water in a beaker. The mixture was stirred at a speed of 480 rpm using a magnetic stirrer for 30 min. Subsequently, 3 g of BC was added to the solution and stirred for an additional 30 min. After that, the pH of the suspension was adjusted to the range of 10–11 by the dropwise addition of a 5 mol·L^−1^ NaOH solution, and the mixture was stirred at 480 rpm for 30 min. Then, the beaker was sealed with parafilm and left undisturbed for 24 h to allow sedimentation. The supernatant was then decanted, and the precipitate was collected via vacuum filtration. The obtained solid was dried in an oven at 80 °C for 24 h until a constant weight was achieved. The dried product was gently ground using a mortar and pestle and sieved through a 100-mesh screen, and the obtained substances were subsequently subjected to pyrolysis at 400 °C for 2 h in a tubular muffle furnace under a nitrogen (N_2_) atmosphere. The resulting product, herein referred to as FBC, was stored at room temperature for further use.

### 2.3. Quantitation of PET

PET microfibers used for the adsorption experiments were prepared by artificially cutting prior PET fibers into microfibers and sieving with 200 mesh sieves. The PET dispersion liquid used for the adsorption experiments was prepared by dispersing PET microfibers in the aquatic solution. After the adsorption, the FBC-adsorbed PET was magnetically separated from the liquid phase by a rubidium magnet. The remaining suspension was filtered through a vacuum filtration pump equipped with a pre-weighed 0.45 μm filter membrane. After filtration, the filter membrane containing the PET mixture was dried in an oven at 80 °C for 24 h and subsequently reweighed. The residual mass of PET was determined by subtracting the initial mass of the filter membrane from the total mass of the dried membrane–PET mixture. The removal efficiency (R) and adsorption capacity (qₑ) of FBC towards PET were calculated using Equations (1) and (2) based on the difference between the initial PET concentration and the final PET concentration:

(1) $$R=\frac{\left(C_{0}-C_{t}\right)}{C_{0}}\times 100\%$$

(2) $$q_{e}=\frac{\left(C_{0}-C_{t}\right)\times V}{W}$$

C_0_ and Cₜ (mg/L) represent the concentrations of PET in solution at 0 h and t h, respectively; qₑ (mg/g) denotes the amount of PET adsorbed per gram of FBC; V (L) is the volume of solution used in each experiment, and W (g) is the mass of FBC applied. To minimize experimental errors and enhance data reliability, all experiments performed in this study were conducted in triplicate.

### 2.4. Batch Adsorption Experiments

#### 2.4.1. Effect of FBC Dosage on PET Adsorption

To investigate the effect of FBC dosages on the adsorption, 0.02 g of PET (1 g/L) was dispersed into 20 mL of deionized water, and a certain amount of FBC was accurately weighed into the PET dispersion liquid. The dosage of FBC was set to 0.1–5 g/L. The prepared samples were placed on a shaker and agitated at a speed of 150 rpm at 25 °C for 24 h. After adsorption, the FBC with PET adsorbed was magnetically separated, and the removal efficiency and adsorption capacity of FBC for PET were then calculated. Based on the results, the FBC dosage yielding the highest removal efficiency was selected for subsequent experiments.

#### 2.4.2. Effect of pH on PET Adsorption

To evaluate the effect of solution pH on the adsorption, the pH of PET solutions (1 g/L) was adjusted to 4.0~9.0, and the FBC dosage was set to 3 g/L. The samples were placed on a shaker and agitated at 150 rpm at 25 °C for 24 h. Based on the calculated removal efficiency and adsorption capacity, the pH condition yielding the highest PET removal was identified and subsequently adopted for the following experiments.

#### 2.4.3. Adsorption Isotherms

Adsorption isotherm experiments were studied with an initial PET concentration that varied from 0.05 to 5 g/L. For each sample, the pH was adjusted to 8.0, and the FBC dosage was set to 3 g/L. To launch the adsorption, the samples were agitated in a shaker at 150 rpm and 25 °C for 24 h. In order to elucidate the adsorption capacity and the relevant mechanism of FBC on PET, the adsorption data were fitted using the Langmuir, Freundlich, and Temkin isotherm models (these models are given in the Supporting Information, Text S1) [43,44,45].

#### 2.4.4. Adsorption Kinetics

For kinetics study, 3 g/L of FBC was added in PET solution (1 g/L, pH = 8.0), and the reaction time was set to 0.083~48 h. The mixtures were agitated in a shaker at 150 rpm and 25 °C for the corresponding reaction time, and the removal efficiency and adsorption capacity of FBC towards PET were subsequently calculated after adsorption. The kinetics data were fitted into the pseudo-first-order kinetic (PFO), pseudo-second-order kinetic (PSO), and Elovich models (these models are given in the Supporting Information, Text S1) [46,47].

### 2.5. Characterization Methods

The surface morphologies of PET, FBC before and after adsorption, and the regenerated FBC were characterized using scanning electron microscopy coupled with energy-dispersive X-ray spectroscopy (SEM-EDS). The surface functional groups of PET and FBC before and after adsorption were identified via Fourier-transform infrared spectroscopy (FTIR). The elemental composition and valence states on the surface of FBC prior to and after adsorption were determined by X-ray photoelectron spectroscopy (XPS), and the crystalline structure of FBC was examined using X-ray diffraction (XRD). The specific surface area and pore size distribution of FBC were measured using the BET method.

### 2.6. Regeneration Performance of FBC

As an adsorbent, FBC should be capable of multiple reuses to minimize investment costs and reduce the time required for preliminary operations. In this study, a cyclic regeneration experiment was conducted to evaluate the reusability of FBC in PET adsorption through four consecutive adsorption–regeneration cycles. The regeneration procedure was as follows: The FBC after adsorption was dried in an oven at 80 °C for 24 h to ensure mass stabilization and was subsequently transferred to a tubular muffle furnace for the pyrolyzation at 400 °C for 2 h (under a N_2_ atmosphere). After pyrolysis, the material was alternately washed several times with deionized water and ethanol to remove soluble organic impurities that could affect the subsequent performance evaluation. After washing, the material was filtered, dried, and stored at room temperature to obtain the regenerated FBC. To evaluate the adsorption performance of the regenerated material, 3 g/L of the regenerated FBC was added to 1 g/L of PET solution with a pH of 8.0. The samples were shaken at 150 r/min and 25 °C for 24 h. After that, the removal efficiency and adsorption capacity of the regenerated FBC towards PET were calculated.

## 3. Results and Discussion

### 3.1. Characterization of FBC and PET

**Figure 1:**
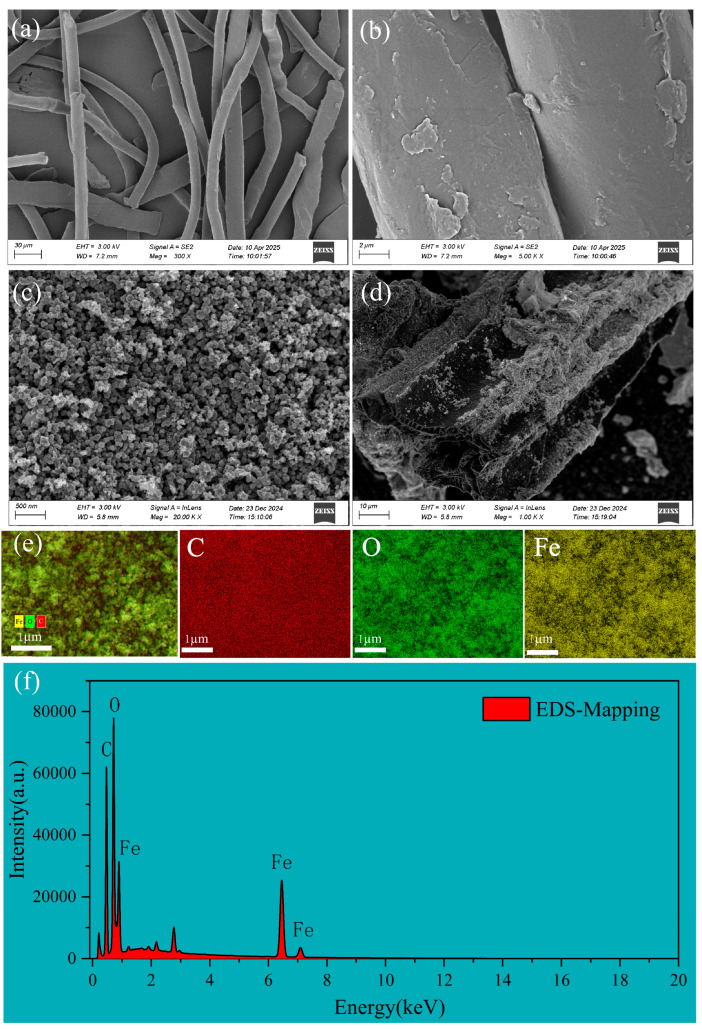
(**a**,**b**) SEM image showing the surface morphology of PET. (**c**,**d**) SEM image showing the surface morphology of FBC. (**e**) Layered EDS elemental mapping. (**f**) EDS elemental mapping spectrum showing the total distribution of C, O, and Fe.

The SEM images of PET indicated a uniform fibrous structure with a relatively smooth surface of PET microfibers, devoid of noticeable cracks or pores (Figure 1a), and the high magnification image further revealed microstructural irregularities on the PET surface, which may be attributed to heterogeneous distribution between crystalline and amorphous regions (Figure 1b).

Some functional groups were observed in the FTIR spectra of PET (Figure S2c). A weak absorption peak at 3432 cm^−1^ corresponded to the stretching vibration of –OH. The peaks at 2915 cm^−1^ and 2964 cm^−1^ were attributed to the C–H stretching vibrations of –CH_2_ and –CH_3_ groups. The strong absorption peak at 1715 cm^−1^ corresponded to the C=O stretching vibration in –COO–, which was one of the most representative structural features of the PET molecule, indicating the presence of ester bonds in its main chain. The absorption band at 1577 cm^−1^ was due to the C=C stretching vibration of the benzene ring backbone. In the mid-to-low wavenumber region, absorption peaks at 1461 cm^−1^, 1409 cm^−1^, and 1344 cm^−1^ were attributed to the bending vibrations of –CH_2_– in gauche and anti-gauche configurations, and the out-of-plane rocking vibrations of –CH_2_– in trans and gauche configurations. The peaks at 1245 cm^−1^ and 1092 cm^−1^ corresponded to the asymmetric and symmetric stretching vibrations of C–O–C. Additionally, the peak at 1009 cm^−1^ was associated with the in-plane bending vibration of the benzene ring, while the peaks at 872 cm^−1^ and 724 cm^−1^ corresponded to the out-of-plane bending vibrations of the benzene ring [48,49]. In summary, PET contains a significant amount of ester bonds and aromatic rings, exhibiting strong hydrophobicity and π-π conjugation characteristics, providing reaction sites and interaction bases for subsequent adsorption with FBC.

The N_2_ adsorption–desorption isotherms and pore size distribution of FBC are presented in the Supporting Information, Figure S1. As shown, the BET-specific surface area of the FBC sample was determined to be 536.04 m^2^/g, and the Barrett–Joyner–Halenda (BJH) average pore diameter was approximately 7.98 nm (based on the desorption branch), with a total pore volume of 0.42 cm^3^/g. These results indicate that FBC exhibits the characteristics of a typical mesoporous material. Given that the PET particles used in this study were approximately 15 µm in diameter—more than 2000 times larger than the average pore size—these mesopores are not directly accessible for PET adsorption. Nevertheless, the mesoporous structure contributes to a high specific surface area, which facilitates the dispersion and surface exposure of iron-containing components on the FBC surface. These exposed components are capable of interacting chemically with PET, thereby indirectly enhancing the overall adsorption performance through surface-mediated interactions.

The SEM images of FBC are given in Figure 1. The FBC particles appeared relatively uniform, with a distinct pore structure and a rough, porous surface, and the nano-sized particles were evenly loaded on the surface of the biochar (Figure 1a,b). The EDS mapping results indicated that C, O, and Fe elements were uniformly distributed across the nanoparticles (Figure 1e,f), suggesting the efficient loading of iron oxide components onto the FBC surface. The diffraction peaks corresponding to Fe_3_O_4_ and Fe_2_O_3_ were observed on the XRD patterns (Figure S2c), suggesting that the loaded iron-based oxides were magnetic Fe_3_O_4_ and Fe_2_O_3_. Additionally, the diffraction peaks were relatively narrow and intense, indicating that the crystallinity of the magnetic particles is well developed [50].

The FTIR spectra of FBC and the original BC are shown in the Supporting Information, Figure S2a. The intensity of the peak corresponding to –OH stretching vibration at approximately 3425 cm^−1^ was weaker for FBC than that for BC, indicating the change in the hydroxyl group during the modification process. The C=C peak at 1604 cm^−1^ shifted slightly downwards, possibly due to the introduction of iron-based components, since the interaction between Fe particles and the π electrons on the carbon material surface could reduce the electron density of the C=C bond, thereby resulting in a lower vibration frequency [51]. Additionally, the conjugation of some aromatic structures may have been disrupted by the metal loading, leading to a further redshift of the absorption peak [52,53]. The C–O absorption peak at 1235 cm^−1^ decreased, indicating a reduction in the number of oxygen-containing functional groups such as carboxyl or ether bonds. At the same time, a distinct Fe–O stretching vibration absorption peak was observed at 583 cm^−1^, confirming the successful loading of iron oxides onto the biochar surface [54]. These results suggested that the number of oxygen-containing functional groups such as hydroxyl, carboxyl, and carbonyl on the surface of BC were decreased during the decarboxylation and dihydroxylation process, resulting in a reduction in surface polarity and hydrophilicity [55]. Such a reduction may weaken the interactions with polar contaminants but could enhance its affinity for hydrophobic pollutants.

The XPS analysis of FBC is presented in the Supporting Information, Figure S3, revealing the elemental composition and types of functional groups on the material surface. The full survey spectrum of FBC (Figure S3a) indicated the presence of C, O, N, and Fe elements on FBC. The high-resolution spectrum of C 1s was deconvoluted into three main peaks: C–C (284.80 eV), C–O (286.36 eV), and C=O (288.83 eV) (Figure S3b) [56], indicating that a certain amount of oxygen-containing functional groups, such as hydroxyl, carbonyl, and ether groups, was present on the surface of biochar composites after high-temperature pyrolysis and magnetic modification. These groups are capable of providing polar binding sites during subsequent adsorption processes. The N 1s spectrum displays three characteristic nitrogen species: pyridinic N (399.77 eV), pyrrolic N (400.71 eV), and graphitic N (401.63 eV) (Figure S3c), and these nitrogen functionalities could enhance the surface reactivity and electron density of the material, thereby potentially increasing its binding affinity for pollutants [57,58]. For the Fe 2p spectrum (Figure S3d), two sets of characteristic peaks were observed, assigning to Fe^3+^ 2p_3/2_ (712.04 eV) and Fe^3+^ 2p_1/2_ (725.13 eV), as well as Fe^2+^ 2p_3/2_ (710.66 eV) and Fe^2+^ 2p_1/2_ (723.86 eV), along with several satellite peaks, confirming the presence of magnetic iron oxides such as Fe_3_O_4_ or Fe_2_O_3_. The presence of Fe^2+^ peaks further indicated a mixed-valence state of Fe^2+^/Fe^3+^ within the FBC, providing additional evidence for the successful incorporation of magnetic components [59]. The O 1s spectrum (Figure S3e) can be deconvoluted into lattice oxygen (O^2−^) from metal oxides (530.37 eV), C–O (531.64 eV), and C=O (533.35 eV), suggesting the co-existence of inorganic oxygen species from metal oxides and organic oxygen-containing functional groups on the FBC surface [60]. Overall, the FBC material not only possesses a large specific surface area and a well-developed porous structure but also exhibits incorporation of Fe–O bonds and abundant oxygen-containing functional groups. The complex chemical surface structure of FBC provides an environment in which a variety of interactions could take place during the adsorption of PET, including π-π interactions, hydrogen bonding, electrostatic attraction, and metal coordination.

### 3.2. Effect of FBC Dosage on PET Adsorption

**Figure 2:**
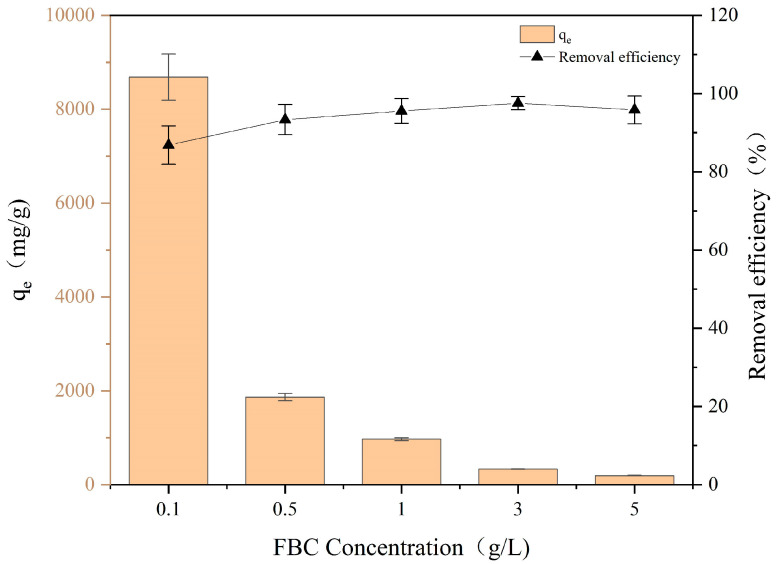
Adsorption capacity of different concentrations of FBC for PET.

Figure 2 shows the change in the removal efficiency and adsorption capacity of PET in a solution with increasing FBC dosage. It can be observed that the removal efficiency of PET gradually rose with the increasing FBC dosage and stabilized at 95.6% when the dosage of FBC exceeded 1 g/L. The removal efficiency of PET increased relatively quickly between 0.1 and 1 g/L but showed only a slight improvement (ranging from 95.6% to 97.6%) when the dosage exceeded 1 g/L. When the dosage exceeded 3 g/L, the removal efficiency changed only slightly (ranging from 95.8% to 97.6%), while the adsorption capacity decreased from about 330 mg/g to about 200 mg/g. Based on the removal efficiency and adsorption capacity of FBC for PET, a dosage of 3 g/L was selected for subsequent experiments under varying conditions.

### 3.3. Effect of Solution PH on PET Adsorption

**Figure 3:**
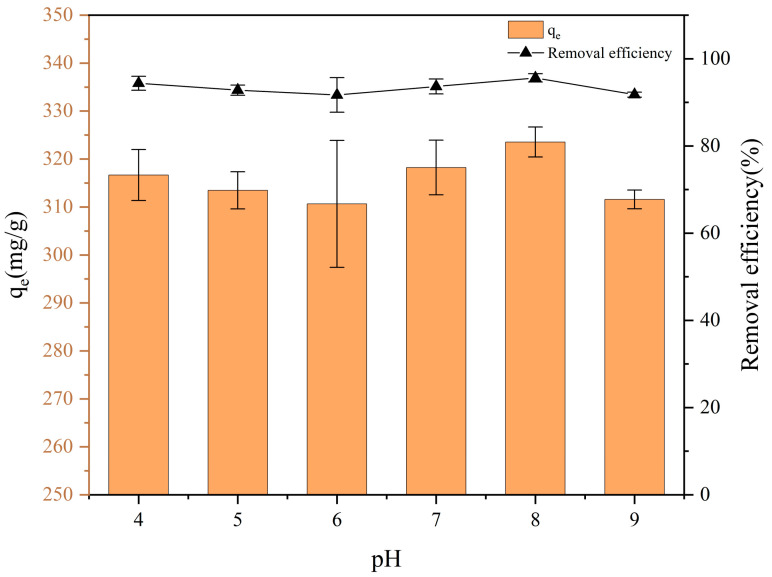
Adsorption capacity of FBC for PET at different pH levels.

As shown in Figure 3, the removal efficiency and adsorption capacity of FBC for PET were observed to exhibit no significant dependence on the solution pH. Within the pH range of 4.0 to 9.0, no substantial variation in either parameter was detected. For subsequent experiments, pH 8.0 was selected as the optimal condition because it is close to neutral conditions, under which FBC remained relatively stable and showed slightly better removal efficiency for PET compared to other pH values.

Variations in the pH of the aqueous environment could alter the protonation state of functional groups on the PET surface, consequently modifying the distribution and magnitude of surface charge density, which may influence the adsorption process [61,62]. However, no significant difference in the removal efficiency or adsorption capacity of FBC for PET was observed with the pH range of 4.0~9.0. This may be related to the porous structure reducing the polarity and enhancing the hydrophobicity of FBC, which led to the occurrence of hydrophobic interactions, π-π interactions, and van der Waals forces among the hydrophobicity and negatively charged PET surface and FBC [62,63]. In addition, the Fe-O component on the FBC is able to form stable metal–O–PET bonds with the PET, which are not easily affected by the pH value [64].

Consequently, the adsorption capacity and removal efficiency of PET remain relatively stable over a broad pH range. This observation is consistent with the findings of Singh et al. [65], where iron-modified biochar was prepared to adsorb carboxylate and amine-functionalized MPs with distinct surface groups. Their experiments demonstrated that for microplastics of the same particle size (1000 nm), removal efficiency varied significantly depending on the functional groups, while negligible effects from interfering ions or pH variations were observed.

### 3.4. Adsorption Isotherms

**Figure 4:**
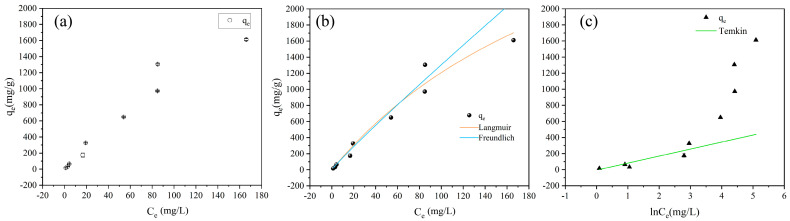
(**a**) Effect of equilibrium PET concentration on the adsorption of PET by FBC; (**b**) fitting curves of the Langmuir and Freundlich models; (**c**) fitting curves of the Temkin models.

The adsorption capacities of FBC for PET under different initial PET concentrations are given in Figure 4a. The data points exhibited a clear upward trend, with the adsorption capacity increasing progressively as the initial PET concentration rose. The adsorption capacity increased rapidly at low PET concentrations, which then gradually leveled off.

The experimental data were fitted to both the Langmuir and Freundlich models (Figure 4b), with the fitting parameters listed in Table 1. The results indicated that the Langmuir model predicted a saturation adsorption capacity of 4500 mg/g, demonstrating the high adsorption capacity of FBC for PET. The separation factor R_L,_ defined by Weber et al. [66], as a dimensionless constant, was applied to evaluate adsorption behavior: R_L_ > 1 indicates unfavorable adsorption, R_L_ = 1 corresponds to a linear relationship, 0 < R_L_ < 1 signifies favorable adsorption, and R_L_ = 0 represents irreversible adsorption [66,67]. Substituting K_L_ = 0.0037 and C_0_ = 1000 mg/L into Equation (S2) yields an R_L_ value of 0.21, which lies between 0 and 1 and approaches 0. These results confirmed that the adsorption of PET by FBC was highly favorable under the experimental conditions, exhibiting good thermodynamic feasibility. The lower R_L_ value further reflected a strong affinity between FBC and PET, with the adsorption process tending to reach equilibrium rapidly.

The fitting performances of the Langmuir and Freundlich models were compared, and the results indicated that both models provided satisfactory fits to the experimental data (Langmuir: R^2^ = 0.9743; Freundlich: R^2^ = 0.9560), with the Langmuir model demonstrating superior performance. This suggests that the adsorption of PET onto FBC tends to follow monolayer chemisorption behavior. The Freundlich parameter K_F_, which represents adsorption capacity, is positively correlated with the adsorbent loading. A higher K_F_ value indicates the superior adsorption capability of FBC. The dimensionless parameter 1/n lies between 0 and 1, indicating favorable adsorption; 1/n > 1 signifies unfavorable adsorption, and 1/n = 1 corresponds to irreversible adsorption [67,68]. In this study, the value of 1/n was observed within the range of 0–1, indicating a moderately favorable adsorption process, which was consistent with the results derived from the Freundlich model. Additionally, the experimental data were fitted to the Temkin model (Figure 4c), yielding poor fitting performance (R^2^ = 0.6560). The Temkin model is generally employed to describe chemisorption processes dominated by electrostatic interactions. This finding suggests that electrostatic forces are not the primary driving mechanism for PET adsorption onto FBC, and the adsorption behavior may involve multilayer physisorption.

Based on the fitting results of the three models, the good fit with the Langmuir model suggests that chemical interactions dominate the adsorption mechanism. Meanwhile, the Freundlich model’s compatibility points to the involvement of multilayer adsorption on heterogeneous surfaces, potentially influenced by van der Waals forces or hydrophobic interactions. These findings indicate that both chemisorption and physisorption may contribute to the overall adsorption behavior of PET onto FBC, with chemisorption being the predominant mechanism under the studied conditions.

### 3.5. Adsorption Kinetics

**Figure 5:**
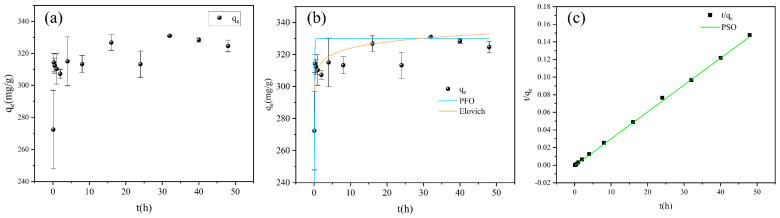
(**a**) Adsorption capacity of FBC for PET at different reaction times; (**b**) fitting curves of the PFO and Elovich models; (**c**) fitting curves of the PSO models.

The adsorption kinetics results and corresponding fitting curves are presented in Figure 5, with the parameters of the PFO, PSO, and Elovich models summarized in Table 2. The adsorption capacity of FBC for PET was observed to increase progressively with time, exhibiting rapid growth within the first hour. As the reaction proceeded, the removal efficiency gradually slowed and stabilized within 24 h (Figure 5a). The PFO, Elovich, and PSO models were employed to fit the kinetic data (Figure 5b,c).

The results demonstrated that the PSO model provided the best description of the adsorption process (R^2^ = 0.9974), yielding an equilibrium adsorption capacity of 328 mg/g. This suggests that chemisorption serves as the primary efficiency-limiting step in the adsorption of PET onto FBC [69]. The adsorption capacity of FBC for PET increased rapidly during the initial stage of the reaction, followed by a deceleration in the second stage, ultimately reaching equilibrium. At the initial stage, bulk diffusion dominated the process, with PET traversing the boundary layer within 1 h. The adsorption process was enhanced by electrostatic attraction between negatively charged PET and positively charged adsorption sites on the FBC surface, as well as the formation of metal–O–PET bonds between PET and metal oxides present on the FBC surface. These interactions collectively facilitated rapid PET diffusion from the aqueous phase to the FBC surface. The second stage is characterized by slow adsorption, where PET becomes entangled with the FBC due to its fibrous structure, resulting in a deceleration in the rate of adsorption capacity increase. Ultimately, adsorption equilibrium is gradually achieved, attributed to the further decrease in diffusion efficiency caused by the reduction in PET concentration in the solution, leading to a stable adsorption state [70].

The PFO model, originally proposed by Lagergren in 1898, is commonly used to describe the adsorption kinetics in solid–liquid systems under non-equilibrium conditions. This model assumes a linear relationship between reactant concentration and reaction efficiency and is typically applicable when the adsorbent possesses limited active sites or when few adsorbates interact with these sites. In certain cases, the PFO model may also represent external or internal diffusion processes. Conversely, if experimental data are more accurately fitted by the PSO model, this indicates that the adsorbent contains abundant active sites and that chemisorption is likely to be the dominant adsorption mechanism. The Elovich model is based on the assumptions of time-dependent increases in activation energy and heterogeneous adsorbent surfaces. Previous studies have shown that the PSO model is generally more suitable than the PFO model for describing the adsorption kinetics of microplastics [71,72]. In this study, the adsorption of PET onto FBC is best described by the PSO model, suggesting that chemical interactions may play a significant role in the adsorption process.

### 3.6. Proposed Adsorption Mechanisms

**Figure 6:**
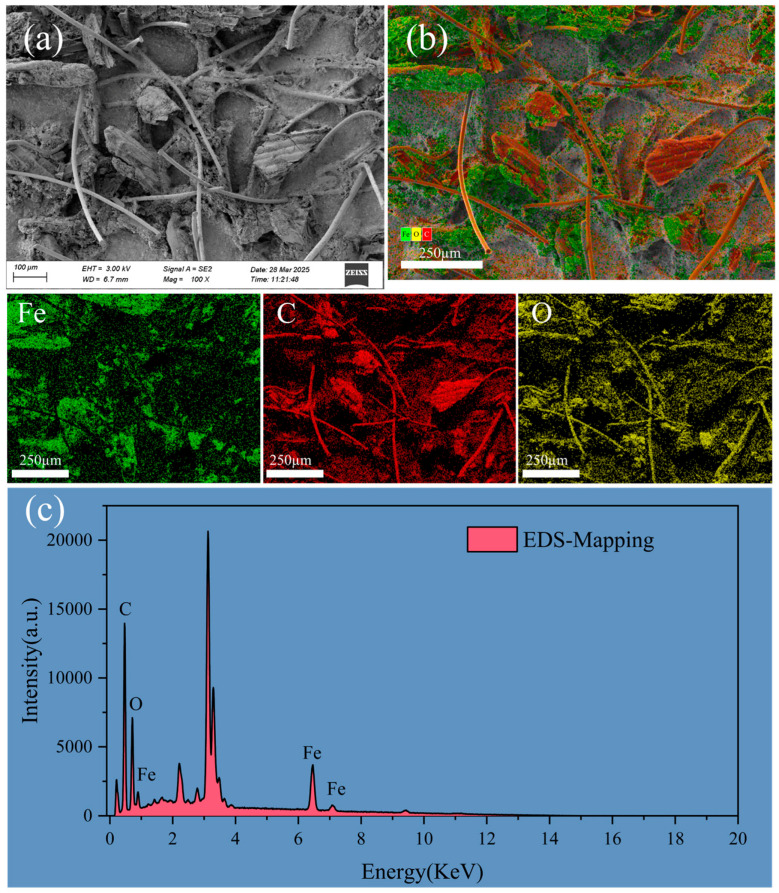
(**a**) SEM images of the mixture of FBC and PET after the reaction; (**b**) Layered EDS elemental mapping; (**c**) EDS elemental mapping spectrum showing the total distribution of C, O, and Fe.

**Figure 7:**
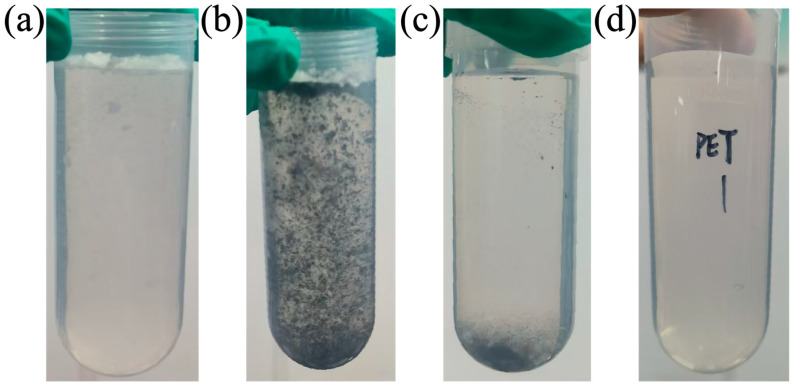
Visual changes during the adsorption process of PET by FBC. (**a**) PET suspension; (**b**) initial mixture of FBC and PET; (**c**) mixture after 24 h of shaking; (**d**) supernatant after magnetic separation.

The SEM images of FBC after adsorption are given in Figure 6. The PET can be observed firmly embedded within the surface folds of FBC. The porous structure of FBC provides a large specific surface area, enhancing the contact area with PET. Notably, the fibrous structure of PET allows it to intertwine with itself or with the surface of FBC during the adsorption process, which could further improve the removal efficiency of PET by FBC. Figure 6c shows the EDS spectrum of FBC after adsorption. The elemental mapping results clearly confirm the presence of Fe, C, and O elements on the surface of FBC. In addition, a distinct peak is observed at approximately 3.2–3.3 keV, which corresponds to the Kα line of potassium (K) according to the X-ray Transition Energies database from NIST. It is noteworthy that K-lines were used in the EDS analysis, which may introduce spectral interference. Nevertheless, the presence of potassium is reasonable, as K is a naturally occurring element commonly found in biomass-derived carbon materials. Similar findings have been reported in previous studies [73,74,75]. The adsorption process of PET onto FBC is illustrated in Figure 7. Figure 7a depicts the dispersed PET solution prior to adsorption; Figure 7b shows the mixture immediately after FBC addition (t = 0); Figure 7c presents the mixture after 24 h of adsorption; and Figure 7d displays the remaining solution following magnetic separation. As observed in Figure 7, FBC retained strong magnetic separability after PET adsorption, with nearly all suspended PET removed from the solution post-separation.

**Figure 8:**
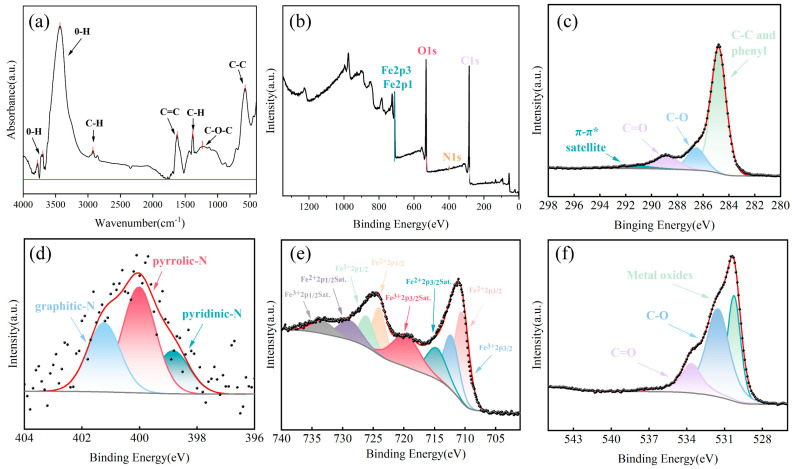
(**a**) FTIR spectra of FBC after the reaction; (**b**–**f**) XPS spectra of FBC after the reaction; (**b**) survey spectrum; (**c**) high-resolution C 1s spectrum; (**d**) high-resolution N 1s spectrum; (**e**) high-resolution Fe 2p spectrum; (**f**) high-resolution O 1s spectrum.The black dots represent the experimental data, and the red lines indicate the fitted curves.

The functional group structure of FBC after PET adsorption is shown in Figure 8a. An absorption peak observed around 3431 cm^−1^ is attributed to–OH stretching vibrations. The relatively high intensity of this peak suggests that hydrogen bonding may have occurred between –OH groups in the FBC and C=O groups in the PET, which might contribute to PET adsorption. However, the weak peak intensity implies limited hydrogen bonding strength. A weaker absorption peak at 2922 cm^−1^ is assigned to the stretching vibrations of C–H bonds, while the peak at 1625 cm^−1^ corresponded to the C=C stretching vibrations. In the mid-to-low wavenumber region, peaks at 1383 cm^−1^ and 1234 cm^−1^ are associated with C–H bending vibrations and C–O–C stretching vibrations, respectively. Additionally, a low-intensity peak at 575 cm^−1^ is related to Fe–O vibrations.

The XPS analysis (Figure 8b–f) reveals the elemental composition and functional groups on the FBC surface post-adsorption. The survey spectrum (Figure 8b) indicates the presence of C, O, N, and Fe, with binding energies and atomic concentrations provided. The C 1s spectrum (Figure 8c) can be deconvoluted into three main peaks corresponding to C–C (284.80 eV), C–O (286.54 eV), and C=O (288.79 eV), along with a π-π* satellite peak at 291.46 eV [56]. This suggests that a certain quantity of oxygen-containing functional groups, such as carbonyl and ether bonds, remains on the surface of the reacted composite. The π-π* satellite peak is attributed to the aromatic structure of PET and typically appears approximately 6 eV above the C–C peak. The N 1s spectrum (Figure 8d) exhibits three characteristic nitrogen species: pyridinic N (398.83 eV), pyrrolic N (400.02 eV), and graphitic N (401.23 eV). Notably, the proportion of pyridinic N increased from 40% to 49%, while that of pyrrolic N decreased from 40% to 31%. This transformation indicates that pyridinic nitrogen may play a crucial role during the PET adsorption. As a Lewis basic site, the lone pair electrons of pyridinic N are capable of forming Lewis acid–base pairs or hydrogen bonds with carboxyl or ester groups in PET, thereby enhancing the adsorption capacity. This process may stabilize the electronic environment of the pyridinic N, with corresponding XPS signals becoming more concentrated and intense, resulting in an increased fitting area. Furthermore, some active pyrrolic N may have participated in π-π interactions or hydrogen bonding, undergoing structural rearrangement or electronic redistribution to transform into the more stable pyridinic structure. This could further account for the observed increase in pyridinic N and the concomitant decrease in pyrrolic N [76]. In the Fe 2p spectrum (Figure 8e), characteristic peaks corresponding to Fe^3+^ 2p_3_/_2_ (712.28 eV) and Fe^3+^ 2p_1_/_2_ (726.11 eV), as well as Fe^2+^ 2p_3_/_2_ (710.54 eV) and Fe^2+^ 2p_1_/_2_ (724.04 eV), along with multiple satellite peaks, remain observable, confirming the stability of magnetic components on FBC and the good reusability of FBC for adsorption. However, a decrease in the Fe^3+^ components is observed (Fe^3+^ 2p_3/2_ decreasing from 29.46% to 17.70% and Fe^3+^ 2p_1/2_ from 29.83% to 23.07%), accompanied by an increase in Fe^2+^ components (Fe^2+^ 2p_3/2_ increasing from 20.23% to 27.35% and Fe^2+^ 2p_1/2_ from 20.49% to 31.89%). These changes suggest the reduction of Fe^3+^ to Fe^2+^ during the adsorption process, likely driven by interfacial electron transfer between polar functional groups or electron donor groups on the PET surface and the FBC, thereby altering the valence state distribution of iron. This further supports the hypothesis of surface interaction mechanisms during adsorption [59,77,78]. The O 1s spectrum (Figure 8e) shows a reduction in the lattice oxygen (O^2−^, 530.26 eV) content from 55% to 32%, possibly due to PET coverage on the FBC surface, which weakens the lattice oxygen signal. The C–O component (531.49 eV) significantly increased from 23% to 49%, indicating the successful adsorption of the ester bonds and ether groups from PET, significantly altering the composition of organic functional groups on the FBC surface. The proportion of C=O (533.58 eV) decreased slightly, which may be due to the partial shielding of the original carbonyl signals or structural rearrangement. These observations further confirm that chemical interactions involving electron transfer between the C=O and Fe–O groups on the FBC surface and the PET occur during adsorption. Additionally, weaker intermolecular interactions, such as π-π interactions and hydrogen bonding, are also involved in the adsorption process [79].

Based on the comprehensive analysis of material characterization and batch adsorption experiment results, the primary mechanisms of PET adsorption onto FBC have been elucidated. SEM images reveal that FBC possesses a porous structure, while PET exhibits a fibrous morphology. A multilayered adsorption structure is formed through the close entanglement of PET and FBC. XPS and FTIR results indicate that functional groups on the FBC surface, such as hydroxyl groups, can interact with PET via hydrogen bonding, π–π interactions, and other mechanisms. Furthermore, ester and carboxyl groups present on the PET surface are capable of undergoing coordination reactions or electron exchanges with Fe sites on the FBC surface, leading to the partial reduction in Fe. Reactions between functional groups in PET and those on the FBC surface likely resulted in the formation of new chemical bonds. Changes in the relative intensities of C–O and C=O peaks in the spectra further support the occurrence of surface reactions between PET and FBC. Combined with adsorption isotherm and kinetic studies, it may be inferred that, during the initial stages of adsorption, the transfer and binding of PET to the FBC surface are primarily driven by chemical interactions, including the formation of Fe–O–PET chemical bonds. As adsorption proceeds and the concentration of PET on the FBC surface increases, the adsorption mechanism transitions to multilayered physisorption driven by the synergistic effects of hydrogen bonding, π-π interactions, and other forces. The adsorption of PET onto FBC thus involves multiple mechanisms and is a synergistic process governed by both chemical and physical interactions.

Moreover, it should be noted that due to the fibrous nature of PET, physical entanglement between PET fibers, or with FBC, is inevitable during the adsorption process. This entanglement can lead to the formation of net-like structures, which enhance the mechanical stability of the PET–FBC complex and facilitate magnetic separation. Although the contribution of mechanical attachment cannot be completely ruled out, our SEM, FTIR, and XPS characterizations support the existence of stable physical and chemical interactions between PET and FBC. Importantly, after adsorption, FBC loaded with PET can be thermally treated to pyrolyze the retained PET, ensuring complete removal from wastewater and avoiding its transfer to sludge. Therefore, regardless of whether PET is adsorbed via chemical interaction or partially retained via mechanical entanglement, the integrated FBC-based process achieves efficient separation and final elimination of PET from WWTP effluents.

### 3.7. Effect of Co-Existed COD on PET Adsorption

**Figure 9:**
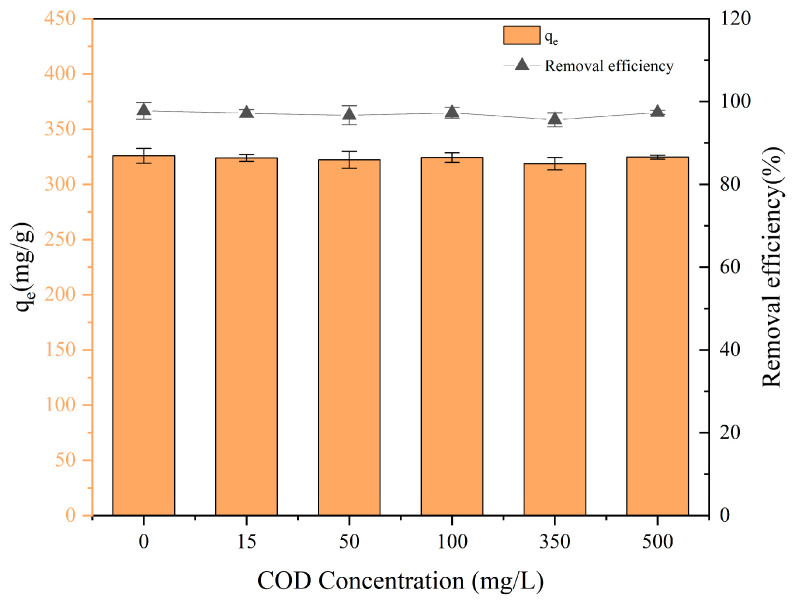
Adsorption capacity of FBC to PET at different co-existed COD concentrations.

Figure 9 illustrates the adsorption capacity of FBC for PET under varying concentrations of co-existed COD (0–500 mg/L). Notably, the adsorption capacity of FBC towards PET remained largely unaffected despite increasing COD levels. In co-existed COD-free conditions, the adsorption capacity reached 326 mg/g. Under low co-existed COD concentration (50 mg/L), this value slightly decreased to 322 mg/g; while medium (350 mg/L) and high co-existed COD concentrations (500 mg/L) resulted in capacities of 319 mg/g and 325 mg/g, respectively. The slight reduction in adsorption capacity can be attributed to the steric hindrance induced by co-existed COD, which may form complexes covering the FBC surface, leading to an increase in negative surface charge and enhanced electrostatic repulsion, thereby reducing the adsorption of PET by FBC. Additionally, co-existed COD may directly compete with PET for the available adsorption sites on the FBC surface, further limiting the adsorption performance [62,64,65]. Nevertheless, within the co-existed COD concentration range of 0–500 mg/L, the removal efficiency only decreased by 2.17%, remaining above 95.59% in all cases, demonstrating that the synthesized FBC exhibits strong resistance to co-existed COD interference and holds promising applicability in municipal wastewater with high co-existed COD levels.

### 3.8. Regeneration Performance of FBC

**Figure 10:**
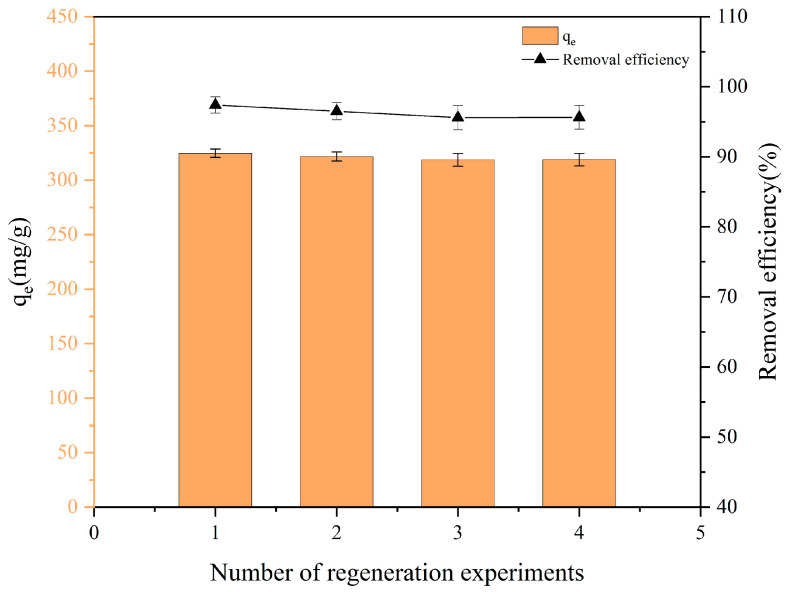
Adsorption capacity of FBC to PET at different number of regeneration experiments.

The removal efficiency and adsorption capacity of FBC for PET after four adsorption–regeneration cycles are presented in Figure 10. The removal efficiency exhibited a progressive decline with successive cycles. The observed reduction can be ascribed to the incomplete elimination of PET-derived residues from the FBC during regeneration protocols. Whilst the FBC underwent pyrolysis at 400 °C under a N_2_ atmosphere, followed by sequential washing cycles with absolute ethanol and deionized water, persistent carbonaceous deposits or thermally induced pyrolytic byproducts were retained on the substrate surface. These residual species are hypothesized to induce pore-blocking effects and/or chemical site occupation, thereby diminishing the availability of active adsorption sites for subsequent contaminant binding. In addition, PET may undergo thermal degradation at around 400 °C to form various byproducts, including acids, monocyclic aromatic hydrocarbons, and alkanes, which could enter the washing solution [80,81]. To minimize the potential environmental impact of the washing effluent generated during regeneration, possible strategies to mitigate this include adsorption using ion exchange resins, advanced oxidation processes, and incineration [82,83,84,85]. These approaches can effectively reduce toxic residues in the effluent and improve the environmental sustainability of the regeneration process. Furthermore, the gradual oxidation of Fe_3_O_4_ on the FBC surface during adsorption reduced the number of available adsorption sites for PET. Nevertheless, as shown in Figure 10, the removal efficiency of FBC remained above 95% even after the fourth regeneration cycle, demonstrating excellent reusability and high removal performance.

**Figure 11:**
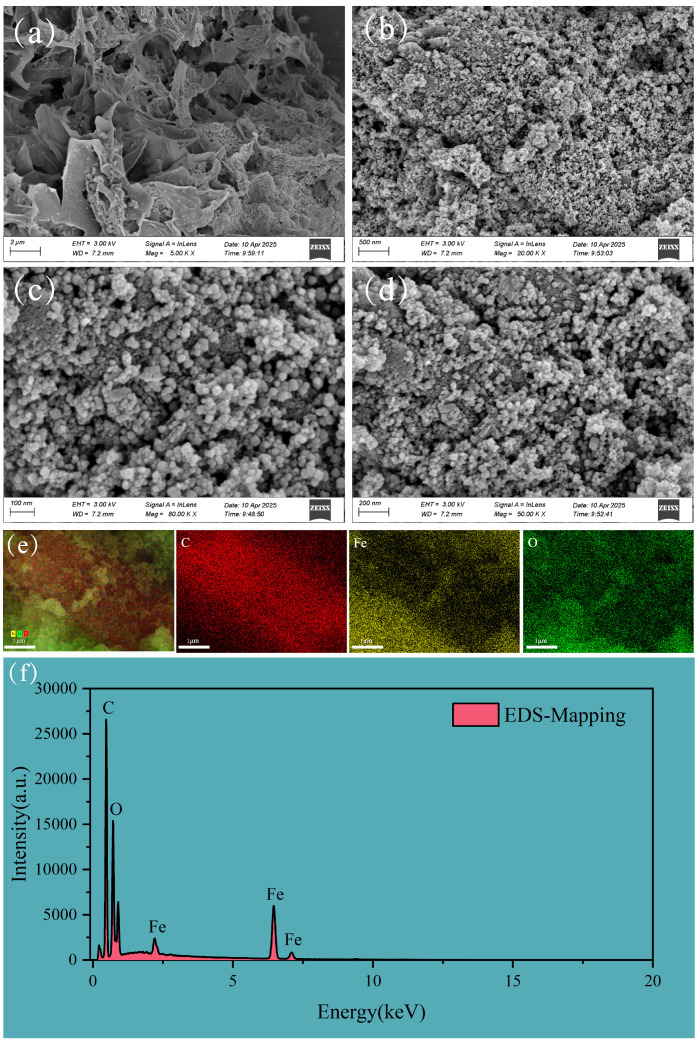
(**a**–**d**) SEM images of FBC after four adsorption–desorption regeneration cycles; (**e**) Layered EDS elemental mapping; (**f**) EDS elemental mapping spectrum showing the total distribution of C, O, and Fe.

The structural stability and adsorption performance evolution of FBC after multiple regeneration cycles are systematically investigated through SEM characterization (Figure 11). Pristine FBC displayed an intact surface morphology with well-developed pore structures, presenting a relatively clean and regular porous network (Figure 1). In contrast, the FBC subjected to four regeneration cycles exhibited a significant increase in surface roughness, along with noticeable particle deposition and partial pore blockage in certain regions, likely attributable to residual contaminants or minor structural degradation during cyclic operations (Figure 11). Nevertheless, the overall porous framework remained well preserved without severe structural collapse, indicating good regeneration tolerance of the material. Even after four regeneration cycles, FBC maintained a high PET removal efficiency, which can be attributed to the excellent structural stability multi-mechanistic synergistic adsorption characteristics. The carbonized framework formed at high temperature remained largely intact during regeneration, continuing to provide abundant adsorption pathways. Additionally, the dispersed iron-based components enhanced the material’s magnetic separation capability and contributed to improved PET capture through electrostatic interactions, coordination bonding, and surface complexation during the adsorption process. Moreover, chemical interactions such as hydrogen bonding and π-π interactions between carboxyl, hydroxyl, and other surface functional groups of FBC and PET were partially retained after regeneration, thereby maintaining adsorption efficiency upon reuse. Overall, FBC demonstrates excellent adsorption performance even after multiple regeneration cycles, highlighting its notable stability and strong potential for reuse. These findings suggest that FBC is an effective and renewable adsorbent with great promise for the removal of MPs [62].

## 4. Conclusions

In this study, a novel nano-sized iron-oxide-loaded biochar (FBC) was synthesized from straw via impregnation–pyrolysis, with magnetization achieved using FeCl_3_·6H_2_O and FeCl_2_·4H_2_O. The adsorption behavior of FBC toward PET microfibers was systematically investigated under varying conditions. The adsorption mechanisms were elucidated based on SEM, XPS, BET, and FTIR characterizations, supported by adsorption modeling. Additionally, cyclic regeneration experiments were conducted to evaluate the reusability of FBC. The main findings are summarized as follows: The optimal adsorption parameters were determined to be an FBC dosage of 3 g/L, a reaction time of 24 h, and a solution pH of 8.0. Under these conditions, the adsorption capacity reached 324 mg/g, and the removal efficiency was 95.6%. Both Langmuir and Freundlich models provided good fits to the experimental data, while the Langmuir model exhibited superior fitting performance. The maximum adsorption capacity predicted by the Langmuir model was 4500 mg/g. Adsorption kinetics studies indicated that the adsorption process was better described by the PSO kinetic model, suggesting that chemisorption played a dominant role. Characterization results indicated that the adsorption mechanism involved the formation of Fe–O–PET chemical bonds, hydrogen bonding, and π-π interactions. Initially, chemical interactions primarily drove the transfer and attachment of PET onto the FBC surface. As the PET concentration increased, the adsorption gradually transitioned towards multilayer physical adsorption driven by hydrogen bonding and π-π interactions. Furthermore, FBC maintained PET removal efficiency above 95.6% across pH 4~9 and COD 15~500 mg/L, confirming the broad applicability of FBC. Notably, FBC maintained a PET removal efficiency above 95.59% after four regeneration cycles. In summary, the FBC synthesized in this study is an efficient adsorbent for the removal of PET microfibers from effluents. Its high adsorption performance and excellent reusability endow it with significant potential for practical application in the treatment of PET-microfiber-contaminated wastewater.

## Figures and Tables

**Table 1:** FBC fitting parameters of adsorption isotherm of PET by FBC.

Langmuir			Freundlich			Temkin		
q_e_ (mg/g)	K_L_	R^2^	n	K_F_	R^2^	lnf	K_T_	R^2^
4500	0.0037	0.9743	1.06	17.23	0.9560	−0.064	87.22	0.6560

**Table 2:** Fitting parameters of adsorption kinetics of PET by FBC.

PFO			PSO			Elovich		
q_e_ (mg/g)	K_1_	R^2^	q_e_ (mg/g)	K_2_	R^2^	α	β	R^2^
330	14.39	0.07711	328	−0.014	0.9974	3.93	0.18	0.7320

## Data Availability

The original contributions presented in this study are included in the article and supplementary material. Further inquiries can be directed to the corresponding author.

## Supplementary Materials

The following supporting information can be downloaded at: https://www.mdpi.com/article/10.3390/nano15120905/s1, Figure S1: N_2_ adsorption–desorption isotherms and BJH pore size distribution of FBC. Figure S2. (a) FTIR spectra comparison of FBC and BC. (b) XRD spectrum of FBC. (c) FTIR spectrum of PET. Figure S3. XPS spectra of FBC and PET mixtures after the reaction. (a) Survey spectrum; (b) High-resolution C 1s spectrum; (c) High-resolution N 1s spectrum; (d) High-resolution Fe 2p spectrum; (e) High-resolution O 1s spectrum.
