# Supplementary material for: Removal of PET Microfibers from Simulated Wastewater Using Magnetic Nano-Ferric-Loaded Biochar: High Adsorption and Regeneration Performance
Source: Nanomaterials (Basel). 2025 Jun 11;15(12):905. doi: 10.3390/nano15120905 (PMC12196203; doi:10.3390/nano15120905)
Supplement: Supplementary file 1 [file nanomaterials-15-00905-s001.zip › nanomaterials-3660135-supplementary.pdf]

Removal of PET Microfibers from Simulated Wastewater using Magnetic Nano Ferric-loaded Biochar: High Adsorption and Regeneration Performance

Beisi Song [1,2], Nini Duan [1,2], Hua Guo Xia [1,2], Yuan Li [1,2*], Hongbin Xu [1,2],

Ying Geng [1,2], Xin Wang [3]

[1] School of Ecology and Environment, Zhengzhou University, Zhengzhou, Henan 450001, PR China

[2] Engineering research center for water emergency response of Henan Province, Zhengzhou, 450001, China

[3] Research Institute of Frontier Science, Southwest Jiaotong University, Chengdu, 610031, China

Supporting Information

TEXTs:

TEXT S1. Models and equations

The adsorption isotherm results were fitted using the Langmuir model, the Freundlich model, and the Temkin adsorption isotherm model. The corresponding model equations are presented in Equations (S1), (S3), and (S4):

Langmuir Isotherm Model:

$$q_e = \frac{q_m K_L C_e}{1+K_L C_e} \tag{S1}$$

$$R_L = \frac{1}{1+k_L C_0} \tag{S2}$$

Corresponding authors

Yuan Li: Tel. +86 0371-67730266; E-mail address: liyuan7626@zzu.edu.cn (Y. Li).

Freundlich Isotherm Model:

$$q_e = K_F {C_e}^{1/n} \tag{S3}$$

Temkin Isotherm Model:

$$q_e = K_T lnC_e + K_T lnf \tag{S4}$$

In these equations, C_0 represents the initial concentration of the PET solution (mg/L), C_e denotes the concentration of PET in solution at adsorption equilibrium (mg/L), and q_e is the amount of PET adsorbed per unit mass of adsorbent at equilibrium under different initial PET concentrations (mg/g). q_m is the maximum adsorption capacity (mg/g) obtained through isotherm model fitting. K_L is the Langmuir adsorption constant, reflecting the adsorption strength or affinity. K_F is the Freundlich constant, indicating adsorption capacity, while n is the Freundlich empirical constant representing adsorption intensity. K_T is the Temkin model constant, associated with adsorption energy, and f is the equilibrium binding constant, which depends on the properties of the adsorbent and temperature.

The adsorption kinetic experimental data were fitted using the pseudo-first-order kinetic (PFO) model, the pseudo-second-order kinetic (PSO) model, and the Elovich model. The respective model equations are provided in Equations (S5), (S6), and (S7):

Pseudo-First-Order Kinetic Model:

$$q_t = q_e(1 - e^{-K_1 \cdot t}) \tag{S5}$$

Pseudo-Second-Order Kinetic Model:

$$q_t = \frac{q_e 2 K_2 t}{q_e K_2 t + 1} \tag{S6}$$

Elovich Model:

$$q_t = \frac{1}{\beta} \ln(\alpha\beta t + 1) \tag{S7}$$

In these equations, q_e and q_t represent the adsorption capacity (mg/g) at equilibrium and at contact time t (h), respectively; K_1 (h^{-1}) and K_2 (g/(mg·h)) are the rate constants for the pseudo-first-order and pseudo-second-order kinetic reactions; α is the initial adsorption rate (g/(mg·h)); β is the desorption constant (g/mg).

Figures:

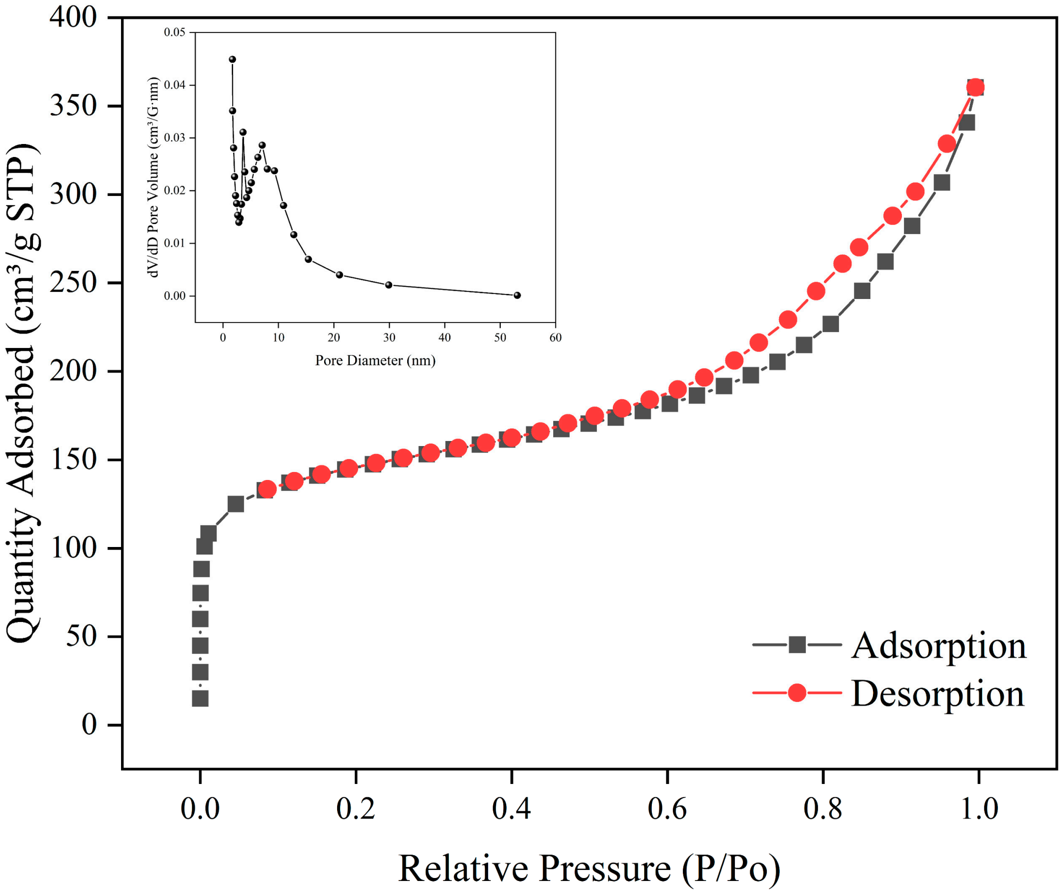

Figure S1. N_2 adsorption–desorption isotherms and BJH pore size distribution of FBC.

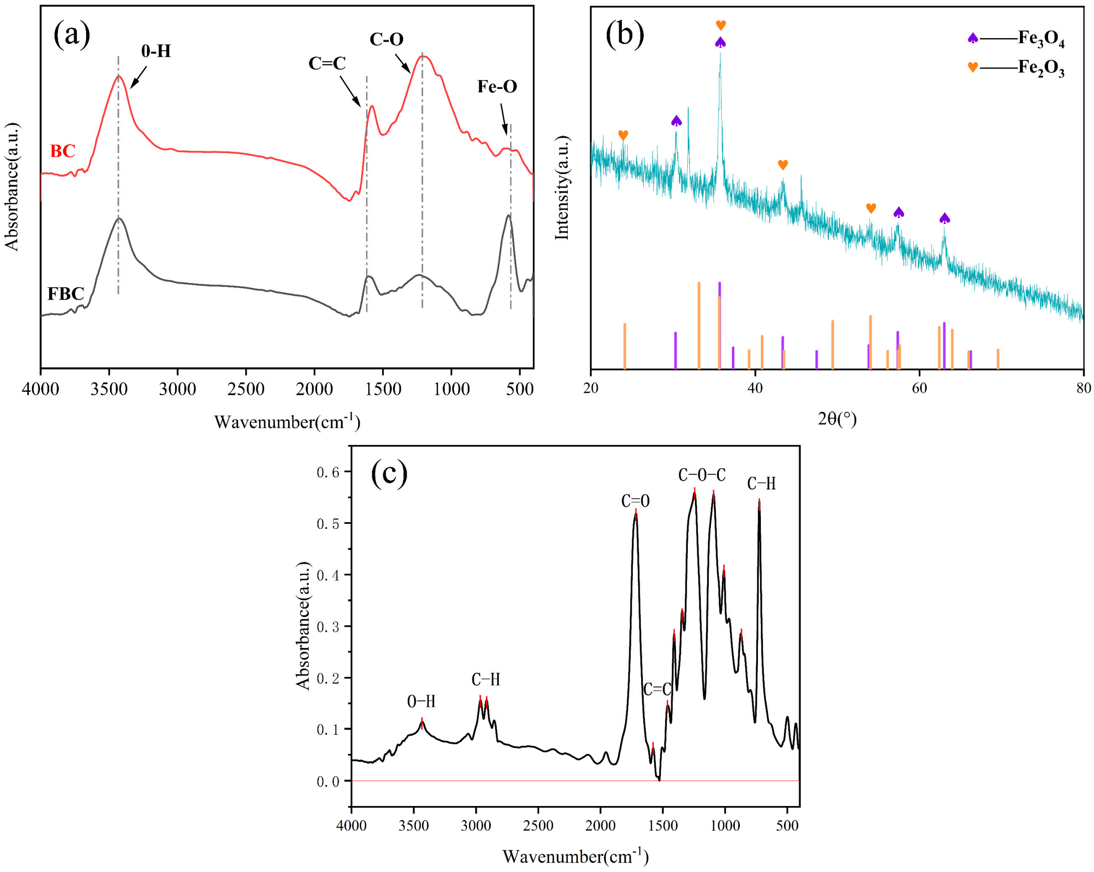

Figure S2. (a)FTIR spectra comparison of FBC and BC. (b)XRD spectrum of FBC. (c) FTIR spectrum of PET

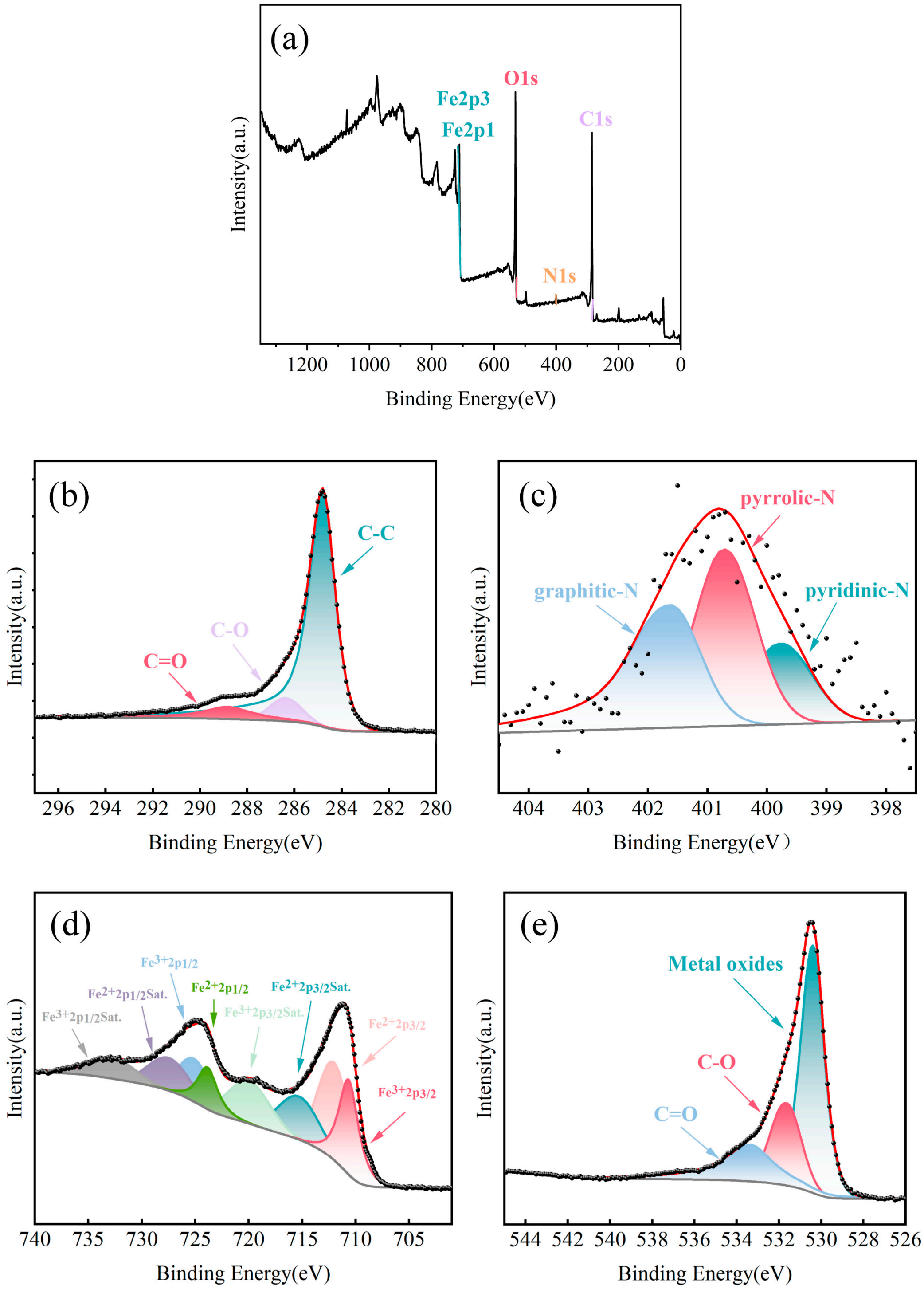

Figure S3. XPS spectra of FBC and PET mixtures after the reaction. (a) Survey spectrum; (b) High-resolution C 1s spectrum; (c) High-resolution N 1s spectrum; (d) High-resolution Fe 2p spectrum; (e) High-resolution O 1s spectrum.
